# Some Tonsil Clippings*Read at Brazos Valley Medical Association Meeting, at Marlin, Texas, May 9, 1899.

**Published:** 1899-07

**Authors:** R. S. Carroll

**Affiliations:** Calvert, Texas


For Texas Medical Journal, Official Organ Brazos Valley Medical Association.

### Some Tonsil Clippings.*

BY R. S. CARROLL, M. D., CALVERT, TEXAS.

### INTRODUCTION.

*Anatomy.*—Man has four tonsils: the two faucal, which are almond shaped bodies, situated between the anterior and posterior pillars of the pharynx; the pharyngeal, or Luschka's tonsil, or so called fourth tonsil, which lies in the base of the pharynx, between the circumvallate papillæ of the tongue and the anterior surface of the epiglottis. In structure they are collections of lymphoid tissue in a more or less compact frame-work of connective tissue, covered with mucous membrane.

*Physiology.*—As understood at present, the office of these bodies is protective and mechanical. Lymphoid tissue is a "germinating center for leucocytes," the lymphoid elements thus combating infections till overpowered by disease. Mechanically, the faucal tonsils elongate the food bolus as it passes and lubricate it with mucous, thus assisting the second act of deglutition. Other theories of their function are unproven. In common with other collections of similar tissue, they gradually atrophy, if not disordered, after maturity.

This paper will treat of four affections of the tonsils, two of which have not received the attention from the general practitioner which their importance merits. I refer to hypertrophied tonsils and adenoids of the naso-pharynx.

Before considering these, however, it may be well to rapidly review those common and sometimes troublesome disorders, follicular and suppurative tonsillitis.

### FOLLICULAR TONSILLITIS.

*Definition.*—Follicular, or more accurately, lacunar tonsillitis, is "essentially an inflammation of the tonsillar crypts, and secondarily of the whole glandular structure."

*Etiology.*—The influence of cold, by temporary resistance of the tissues to the action of the ever present pathogenic germs, is the most frequent cause of this malady. Gastro-intestinal disorders, and the rheumatic diathesis are also causative; while chronic en-

*Read at Brazos Valley Medical Association Meeting, at Marlin, Texas, May 9, 1899.

largements of the tonsils naturally invite recurring attacks. "The disease begins, probably, as an infectious inflammation at the bottom of the crypts, due to the presence of strepto- and staphylococci, which readily enter from the mouth and excite an attack whenever favorable conditions are present."—Holt.

*Symptoms.*—The symptoms are familiar. They come on rapidly,—a chill, pain in the muscles and head, and rapidly rising temperature, which often reaches 104° to 106° within a few hours. The throat becomes more and more uncomfortable, the tonsils swell, and the orifices of their crypts are filled with a friable, cheesy exudate. Both tonsils are usually involved at the same time. The constitutional symptoms improve after the first day, the throat remains painful four or five days, while the swelling persists for a week or longer.

*Diagnosis.*—Any exudation in a child's throat is cause for alarm to parents, and the physician can render true service to the family, as well as to the State, by making an early diagnosis between this affection and diphtheria. Undoubtedly many of the profession are guilty of careless or superficial work in making the distinctions which exist. The clinical points brought out in the following parallel columns are vital in making a clear differentiation:

| DIPHTHERIA. | FOLLICULAR TONSILLITIS. |
|---|---|
| Usually epidemic. | Not epidemic. |
| Onset gradual, temperature 102°, pulse weak, constitutional symptoms mild. | Onset abrupt, temperature 103-6°; and general symptoms severe. |
| Seldom a history of previous attacks. | History of previous attacks not uncommon. |
| Albuminuria the rule. | Albuminuria the exception. |
| Membrane thick, leathery, white or gray; comes away in masses, leaving a bleeding surface. | Membrane thin, friable, yellow, less necrotic in appearance; and comes away in small pieces, leaving no bleeding surface. |
| Membrane usually not limited to the tonsils and spreads rapidly. | Membrane limited to the tonsils, and shows no tendency to spread. |

Paralytic symptoms strongly indicate diphtheria.

Too frequently for the comfort of anxious parents we hear the term "diphtheretic sore throat" used, when the case is simple noninfectious tonsillitis. On the other hand, the welfare of the community is jeopardized by all those cases of "tonsillitis," so-called, which die promptly during the first week of illness. Our best thought is none too good in making the differentiation in these cases.

*Prognosis.*—The chief danger in this form of tonsillitis is that a degree of tonsillar hypertrophy will persist. Life is in danger only in the rarest cases.

*Treatment.*—Abortive treatment is not impracticable, if the case is seen the first day. A thorough application of pure guiacol, while painful, will check the disorder in a large percentage of cases. This agent can be applied by spray or swab to the tonsils and peritonsillar tissue. The bowels should be opened with a brisk saline, aconite in hourly doses to the physiological extent, and in rheumatics, salol in solution should be administered hourly in three to four grain doses. These means will, in most instances, insure rapid resolution. Unfortunately, however, many cases are seen too late to be reached by these means, and we may be content with affording a degree of relief and allowing nature to conduct this self-limited disease. The swollen tonsil may be depleted by several punctures with a keen bistoury, antipyrine will relieve the general discomfort, while a gargle of hot bicarbonate of soda solution will relieve the throat of the thick and disagreeable secretions. In fact, the dry soda may be applied to the tonsil to the great relief of many patients, who will rub it into the organ themselves when they discover the comfort which follows. The old mixture of tincture of iron, one drachm to the ounce of glycerine, taken in teaspoonful doses, without water, every one or two hours, is satisfactory routine treatment. Guaiac in its various forms is a favorite with many, but will not prove the specific that many claim it to be. Aconite is especially happy in its effects on children. Pellets of ice are often most agreeable.

PERITONSILLAR ABSCESS—SUPPURATIVE TONSILLITIS—QUINSY.

*Definition.*—Peritonsillar abscess is a true phlegmon, situated in the connective tissue surrounding the tonsil, in the course of which the tonsil suffers by contiguity.

*Etiology.*—The disorder is most common during the second and third decades of life, probably because it is during this period that hypertrophied tonsils are most frequent, for the diseased condition of the enlarged organs seems to invite this distressing malady. All other general and local causes which are factors in producing acute follicular tonsillitis influence this condition as well.

*Symptoms.*—The general symptoms of the follicular variety are also found here, though intensified. Locally, the conditions differ. The disturbance is usually unilateral, the tonsil itself does not take active part in the reaction, but the tissues immediately surrounding are swollen, often edematous, intensely red and literally as painful as a boil. Articulation is hampered, though phonation is fairly clear. The tongue is foul, the breath miserably offensive. Mucus and saliva accumulate and are slowly and painfully hawked out or droll from the mouth, for deglutition is agonizing and at times so difficult that fluids will be allowed to regurgitate through the nose rather than suffered to pass the painful area. At times the uvula may become edematous, and in those rare instances when the affection is bilateral, breathing may be interfered with. No local disease so rapidly exhausts the patient as this. Sleep, eating and drinking are well nigh impossible.

*Diagnosis.*—The diagnosis is without difficulty. The history of a chill, fever, and pain in the throat are not diagnostic, but the patient's attitude is extremely characteristic: the neck fixed, the head forward, the slow hawking or drolling of saliva; the inability to more than partially separate the jaws, and the fetid breath make a picture of pitiable distress not found in another acute disease.

*Treatment.*—This disorder may also be aborted in a fair percentage of cases if seen early. Guiacol locally, aconite in hourly doses till its effect is evidenced by tingling of the tongue or fingers, and full doses of salicylate of soda in all cases in which any rheumatic tendency is discernable, are the chief measures in which reliance may be placed to cut short an attack. If this desirable end cannot be attained, the case should be handled very much as the follicular form. Salol in generous hourly doses, bicarbonate of soda locally to assist in the removal of the viscid secretions, and the antipyretics are indicated. Several deep stabs into the tonsil often afford considerable relief through the abstraction of blood. The tumor should be carefully watched for fluctuation, which is detected much earlier by palpation than inspection. As soon as the presence of pus is assured, a free opening should be made, not—as is so frequently done—into the tonsils proper, but into the most bulging point of the peritonsillar tissue. Immediate relief follows the abstraction of pus, whether operative or spontaneous. After resolution the enlarged tonsil should be removed, as this almost without exception prevents recurrences.

HYPERTROPHIC TONSILLITIS—CHRONIC TONSILLITIS.

*Definition.*—Hypertrophic tonsillitis is a chronic enlargement of the tonsil, consisting of a hyperplasia of both lymphoid and connective tissue elements, with a dilatation and clogging up of the crypts with waste matter. This is a condition which constantly invites fresh attacks of inflammation. It is to be remembered that "when any morbid process fixes itself on lymphoid tissue it is prone to remain, and moreover, produce permanent hypertrophy." Furthermore, normal lymphoid tissue is protective, but hypertrophied tonsils, with their gaping or filth containing crypts are channels open to convey any infection into the system through the lymphatics. Tuberculosis, diphtheria, scarlet fever and other diseases may be and doubtless are received through this portal.

*Etiology.*—Like the majority of tonsillar affections, this is most common during the first two decades of life, though by no means limited to this period. I recall the case of a lady of considerable refinement who, with much embarrassment, sought relief for her fetid breath. She had visited the dentist repeatedly and taken much physic for "indigestion and biliousness," hoping thus to rid herself of her mortifying affliction. Examination disclosed a pair of tonsils about double normal size, quite hard, but with several crypts loaded with foul debris. Relief was simple when the cause was finally discovered. This poor woman stated that for over twenty years this condition had influenced her life. Her husband accused her of coldness, her children and friends considered her unapproachable, and she was miserable whenever near any one because of the consciousness of her disagreeable breath. Preceding attacks of acute tonsillitis and the infectious diseases, with heredity, account for most cases of enlarged tonsils.

*Symptoms.*—"Enlarged tonsils are in a sense foreign bodies and give rise to symptoms accordingly." In babes, nursing may be interfered with and the breathing difficult at times. Later in life the voice is thick or "mushy" in quality, the senses of taste, smell and hearing are usually defective to a degree, and the chest develops poorly. These children are often poor sleepers, subject to "night-terrors." The breath is offensive; in fact, these persons live in a vitiated atmosphere. Every breath they inhale is contaminated in passing these unwholesome bodies, while the bolus of food scrapes masses of necrotic matter from the crypts in passing, which undoubtedly helps to produce the frequent stomach disturbances common to these patients.

*Diagnosis.*—Palpation in babies, and inspection in children and adults, easily reveal the enlarged masses. It is to be remembered that the normal tonsil is not visible when the throat is at rest.

*Prognosis.*—In probably no condition does the advice of the physician so frequently meet with the weak objection, "Well, I believe the Creator put those things in the throat for a purpose, and I don't think they should be bothered." The Lord undoubtedly did "put those things in the throat for a purpose," but he put them in healthy and of a normal size, and no more intended that a human being should exist with a throat full of foul tonsil tissue than he expected man to live with a mouth filled with decayed teeth. How an intelligent person can reconcile the presence of either of these filthy conditions in his own or his child's mouth with ordinary personal decency, to say nothing of health, is an unsolved problem. Children with these enlargements are "more susceptible to diphtheria, scarlet fever and croup, and these diseases are more prone to be fatal in these patients." "Irreparable damage may have been done in various directions before spontaneous atrophy occurs." Therefore, the physician is not justified in waiting for the child to "grow to his tonsils," and should he so advise in a case and that child should contract diphtheria or similar infection, the responsibility of that life would rest heavily on that physician's shoulders.

*Treatment.*—If the tonsils are large they should be thoroughly clipped. It is a waste of time to apply nitrate of silver, iodine or other local preparations, or await the effect of tonics, which never effect. It is a safe rule to consider all visible tonsil tissue at eighteen years to be diseased. In mild cases, or when the crypts are simply dilated, it is sufficient to clean out the crypts and pencil them to the bottom with chromic acid, or better, to use the galvano-cautery. Applications of nitrate of silver are useless, as this drug seems to stimulate the organs to increased growth. The operation is simple, and a few suggestions will cover all cases. Adhesions between the tonsil and the pillars of the pharynx should be separated with a blunt probe before cutting the tonsil. The organ is not especially sensitive, though a little cocaine may be injected if wished. Small or nervous children should receive general anesthesia. For soft tonsils in patients under eighteen years, the bistoury or tonsillotome may be employed, but for older patients, or in operations on indurated organs, the cold wire snare or galvano-cautery loop is undoubtedly safer. Cocaine is then requisite. "Fatal hemorrhage" is the bug-a-boo of the timid doctor. Undoubtedly severe and alarming bleeding has followed amputation of the tonsil, but if the precaution is observed to never cut a hard organ after the age of eighteen, depending on the snares in these cases, serious bleeding will occur in the proportion of about one case in fifty thousand.

Before quitting this subject I wish to refer again to the dense ignorance of the average person on the subject of tonsillar hygiene. Our patients should know that the healthy tonsil is practically invisible; that enlarged tonsils obstruct the air blast through the nose. This allows nasal secretions to accumulate and decompose, inviting catarrh. That chronic sore throat (pharyngitis) in the adult is often the remnants of tonsils the Creator did not put in. Draw their attention to the thick, cloudy, woody, dead voice of their child, the imperfect articulation, the sickening breath and chronically foul tongue. Show them how all the air that goes to the lungs and every swallow of food is contaminated, which auto-intoxication accounts for the sallow skin and general anemia so frequently associated with foul tonsils. And finally insist that in clipping away the diseased portion you are no more desecrating the Lord's handiwork than does the dentist when he draws a hopelessly disordered tooth.

### HYPERTROPHY OF THE PHARYNGEAL TONSIL—ADENOID VEGETATIONS.

*Definition.*—This is a hypertrophy of the lymphoid elements of the pharyngeal tonsil, in every way similar to that which occurs in the faucal tonsil. The condition was first fully described by Meyer, of Copenhagen, in 1868, and is one which has been much neglected by the general practitioner. This is the more remarkable in view of the efforts of specialists to keep the subject prominently before the profession.

*Etiology.*—The hypertrophy develops early in childhood, and shows a tendency to disappear at puberty. Heredity is a marked influence, and all causes which favor the abnormal development of lymphoid tissue are etiological factors in its production. This condition may, and frequently does, exist independent of any enlargement of the faucal tonsils.

*Symptoms.*—One symptom is so characteristic that it should attract the attention of even a superficial diagnostician; this is the habit of mouth breathing. A child with well developed adenoids often presents the appearance of stupidity, with its heavy eyes, half open mouth and dull, expressionless face. The imperfect hearing, which almost always results from the obstruction, adds much to the impression that the child is mentally deficient. As a rule, however, the intellect is normal. But the sensibilities are sure to be blunted by continued abnormal breathing, the constant presence of mucus or scabs in the nostrils, which cannot be thoroughly or at times partially expelled, the constant association with a foul breath and the deficient hearing. The fact that there is a permanent obstruction to the proper ventilation of the middle ear explains the evil effects on the ears, the drums of which are found retracted. The voice is altered, "n," and "m" cannot be pronounced; and stuttering is not a rare accompaniment of the disorder. As a secondary result the chest is poorly developed, even to the extent of "chicken breast" in some cases. These children "always have cold," as the parents suppose, for they cannot dispose of the excessive quantities of mucus which results from the accompanying naso-pharyngitis. As babes they nurse from the breast imperfectly, if at all. As children they sleep poorly, being especially subject to night-mare, which differs from that of indigestion in that the attacks are repeated several times in one night. Associated with all these symptoms is a strong tendency to sore throat, ear-ache, ear discharges, catarrh and bronchitis. All in all, it is a blessing to these children that their sensibilities are blunted, as their lot is truly miserable as it is.

*Diagnosis.*—In a well marked case the diagnosis can hardly be mistaken. The intractable nasal catarrh, the mouth breathing, the characteristic vapid facial expression, the disturbed, snoring sleep all point strongly to this condition. If practicable the rhinoscopic mirror may be used, but the finger is usually more satisfactory. A few folds of a towel will prevent biting, while the finger is slipped behind the palate. In the soft variety the naso-pharynx will be found more or less filled with a mass that feels like a bundle of earth worms, and the finger will be covered with blood when removed, no matter how gentle the manipulations may have been. In other cases the mass will feel like a cushion. As a rule, little can be seen by anterior rhinoscopic examination. Another, though not as certain, means of diagnosis is the use of the oily spray. In the normal nose, when a spray is forced into one nostril it will return through the other in a volume but slightly less than that of the atomiser; but when adenoids are present, this is not the case, and the spray will not return, or at best will come back in greatly lessened volume.

*Prognosis.*—The prognosis is thoroughly unsatisfactory unless this foreign mass is removed, and this cannot be accomplished with any promise of success by other than surgical means. And the results of the operation fully justify it. To quote from "The American Text-book of Diseases of the Eye, Ear, Nose and Throat": "There is no operative procedure in the whole domain of this branch of medicine attended by happier results than is that for the removal of lymphoid hypertrophy from the vault of the pharynx. The child is physically almost born again. Dull intellects brighten, deaf ears are unstopped, phonation becomes clear and distinct, mouth breathing disappears. In short, the child is a new creature." Recurrence after operation is in some cases unavoidable, but seldom do the conditions again become as distressing as at first, and my experience has been that parents are not only willing but are anxious to have the operation repeated, if the symptoms recur, for they have realized the wonderful benefits which follow removal of the vegetations.

*Treatment.*—All means which do not cause the disappearance of the growth are unscientific, and all methods of removal other than active surgical means are disappointing. In children under twelve years general anesthesia should be employed, the mouth-gag inserted, the region thoroughly explored with the finger, and if the mass is very soft much of it can be quickly scraped away with the finger nail. Otherwise, the post-nasal curette, or cutting forceps is necessary. After clearing out the dome of the pharynx it is well to pass a straight curette back through the nostrils and clear out the posterior nares. No after treatment, save an occasional irrigation with warm salt water, is needed for the local condition. The syrup of iodide of iron and cod liver oil assists materially in the return of that health which is childhood's right.

Finally, let us as physicians realize the vast amount of needless suffering that arises from disorders of the naso-pharyngeal lymphoid clusters, and while the only rational treatment is not popular to our patients, let us not risk their future well-being by a weak policy of postponment or briggling therapeutics.

